# Social-Biological Interactions in Oral Disease: A ‘Cells to Society’ View

**DOI:** 10.1371/journal.pone.0146218

**Published:** 2016-01-11

**Authors:** Noha Gomaa, Michael Glogauer, Howard Tenenbaum, Arjumand Siddiqi, Carlos Quiñonez

**Affiliations:** 1 Department of Biological and Diagnostic Sciences, Faculty of Dentistry, University of Toronto, Toronto, Ontario, Canada; 2 Discipline of Dental Public Health, Faculty of Dentistry, University of Toronto, Toronto, Ontario, Canada; 3 Department of Periodontology, Faculty of Dentistry, University of Toronto, Toronto, Ontario, Canada; 4 Discipline of Social and Behavioural Sciences, Dalla Lana Faculty of Public Health, University of Toronto, Toronto, Ontario, Canada; UNC School of Dentistry, University of North Carolina-Chapel Hill, UNITED STATES

## Abstract

Oral diseases constitute a major worldwide public health problem, with their burden concentrating in socially disadvantaged and less affluent groups of the population, resulting in significant oral health inequalities. Biomedical and behavioural approaches have proven relatively ineffective in reducing these inequalities, and have potentially increased the health gap between social groups. Some suggest this stems from a lack of understanding of how the social and psychosocial contexts in which behavioural and biological changes occur influence oral disease. To unravel the pathways through which social factors affect oral health outcomes, a better understanding is thus needed of how the social ‘gets under the skin,’ or becomes embodied, to alter the biological. In this paper, we present the current knowledge on the interplay between social and biological factors in oral disease. We first provide an overview of the process of embodiment in chronic disease and then evaluate the evidence on embodiment in oral disease by reviewing published studies in this area. Results show that, in periodontal disease, income, education and perceived stress are correlated with elevated levels of stress hormones, disrupted immune biomarkers and increased allostatic load. Similarly, socioeconomic position and increased financial stress are related to increased stress hormones and cariogenic bacterial counts in dental caries. Based on these results, we propose a dynamic model depicting social-biological interactions that illustrates potential interdependencies between social and biological factors that lead to poor oral health. This work and the proposed model may aid in developing a better understanding of the causes of oral health inequalities and implicate the importance of addressing the social determinants of oral health in innovating public health interventions.

## Introduction

Oral diseases are some of the most common chronic conditions around the world [1]. Despite the advent of preventive and therapeutic dentistry, oral diseases continue to place a heavy toll on socially disadvantaged groups, creating persistent social gaps in oral health [2]. In Canada, for example, over 95 percent of adults are affected by untreated coronal decay and/or periodontitis, with the burden of disease concentrating in individuals of lower income and education, those who lack dental insurance, and those who decline recommended dental care because of costs [3]. Such inequalities have led several national and international institutions, such as the Public Health Agency of Canada (PHAC), World Health Organization (WHO) and International Association for Dental Research (IADR), to call for a better understanding of the causal pathways in oral disease to inform public health policy and to guide new and innovative public health interventions [4].

From an aetiological stance, periodontal disease has consistently been linked to the interplay between plaque and the host-immune response. Extensive research has shown that while periodontal conditions are initiated by dental plaque, perpetuation of inflammation and the severity and progression of the disease depends upon the effectiveness of the innate immune response to the bacterial biofilm [5–7]. Meanwhile, dental caries is essentially a diet-mediated disease in which host factors, such as immune components in the microbial biofilm and saliva contribute to its progression [8, 9]. While several factors are known to modify variations in these host factors, such as genetic and systemic elements, they cannot explain, on their own, the social differences in oral health [10]. Consequently, addressing these factors alone as the causes of oral disease has resulted in reductionist approaches to prevention and treatment that often lack a sound theoretical basis, and that have generally been unsuccessful in reducing the burden of oral disease and oral health inequalities [11].

Similarly, considerable work to understand the causes of oral disease and related inequalities has focused on the role of behavioral factors such as poor oral hygiene, smoking and alcohol consumption, where poor oral health and related inequalities have been attributed to the lack of health education, especially in socially disadvantaged groups [12, 13]. However, downstream behavioural interventions (e.g. oral health education programs) have proven ineffective in achieving sustainable behavioural change [11, 14–16]. In fact, studies suggest that such interventions have potentially widened the health gap between social groups [14]. This failure has been attributed to ignoring the social, economic and psychosocial environments in which behaviours occur when designing such interventions [10].

Research in social epidemiology has drawn on the biopsychosocial pathway for plausible links between social factors and oral disease. Evidence has consistently shown that chronic psychosocial stressors related to social adversity, such as low socioeconomic position (SEP), material deprivation and poor social relationships, can influence physiological body functioning, as well as behaviour in relation to disease [17]. This has been characterized in studies demonstrating the social patterning of stress hormones and biomarkers in various health conditions such as cardiovascular disease (CVD), obesity, asthma and depression [18–24]. Despite the association between adverse social conditions and health outcomes, a lack of understanding persists as to the psychosocial and biological pathways through which the social becomes embodied, or ‘gets under the skin,’ to bring about disease and ultimately leading to health inequalities. Indeed, Marmot has suggested that deeming a particular psychosocial factor as both a cause of ill-health and a contributor to social inequalities in health requires an understanding of the biological mechanisms involved [25].

In this paper, we aim to understand the mechanisms by which social adversity becomes embodied to interact with biological systems in terms of oral disease. We first provide an overview of “embodiment” and then present a systematic review of the social-biological interplay in oral disease, with the purpose of identifying knowledge gaps in this area. Based on the results of our review, we propose a dynamic model that depicts the putative interdependencies between social and biological factors that lead to poor oral health. To conclude, we shed light on some of the public health policy implications and research opportunities in the field.

## Understanding Embodiment

Several studies have explained the social patterning in health by exploring mechanisms along the biopsychosocial pathway [26–29]. These suggest that health inequalities result from differences in the experience of stress between social groups due to material and psychosocial factors [30]. For example, individuals of lower SEP experience higher levels of stress due to precarious living conditions and the inability to meet daily needs. Similarly, psychosocial factors such as lack of social support, job security and job strain, have been shown to relate to worse health outcomes [31–33]. The resultant stress–both over the life course and at different life stages–becomes embodied by altering the body’s neuroendocrine and immune responses leading to an increased cumulative physiological burden and the initiation and progression of oral disease [32–34]. Thus, in order to understand social-biological interactions and their promotion of poor oral health, it is fundamental that we grasp the pathophysiological mechanisms involved as well as the mediators by which stressors are transduced into the body.

### 2.1. The allostatic load experience

Perhaps one of the most compelling explanations of how social adversity translates into biological processes is that of “allostatic load” [35]. Allostatic load represents the “wear and tear” on the body that results from repeated attempts to maintain homeostasis in response to prolonged stress challenges [36]. When psychological demands exceed an individual’s capacity to adapt physiologically and emotionally, this can have physiological implications that may compound over time and trigger chronic disease [37]. More specifically, the dysregulation of the hypothalamic-pituitary-adrenal (HPA) and the sympathetic-adrenal-medullary (SAM) axes produces stress hormones such as cortisol, epinephrine and norepinephrine, which in turn disrupt the neuroendocrine, immune and metabolic systems [38]. This notion of stress acting as a common risk factor to the dysregulation of various body systems places an emphasis on the interaction between different biological systems in the body and considers the impact of cumulative physiological dysregulation over the life course as a disease risk [39]. Indeed, allostatic load has been shown to increase the risk for a number of health conditions, including CVD, cognitive and physical decline and all-cause mortality [38, 40–42].

A number of parameters have been used to measure allostatic load. McEwen proposed a summative score where markers such as systolic and diastolic blood pressure, waist-to-hip ratio, serum HDL and total cholesterol, glycated haemoglobin, and overnight urinary cortisol secretion can be used as an aggregate score [35, 43]. These measures have been used with the aim of attaining a range of values for markers that include the different regulatory systems involved in adapting to allostatic load [43, 44]. Interestingly, allostatic load has been suggested to have an additional predictive power in disease risk over individual biomarkers and provides a potential understanding of the physiological burden imposed by exposure to detrimental stressors.

Unsurprisingly, allostatic load is socially patterned, with higher allostatic load being associated with social disadvantage. Studies have shown that allostatic load is associated directly with senility, psychological stressors, poor neighbourhood conditions and low SEP [18, 45–47]. Alternatively, healthy social ties as well as higher income and education levels seem to ameliorate the effects of allostatic load [45, 48]. For instance, Kubzansky et al. linked SEP, measured by educational attainment, to psychosocial vulnerability and allostatic load, concluding that lower levels of education and greater levels of hostility are associated with higher allostatic load indices. Alternatively, higher income and education levels as well as healthy social ties have been shown to mitigate the effects of allostatic load [45, 48].

### 2.2. Behaviour as a mediator of stress

Allostatic load not only reflects the influence of social circumstances and stressful life experiences, but also behaviours, such as smoking, diet, exercise and alcohol consumption, which influence the reactivity of physiologic systems to stress [36]. These health behaviours are well-known common risk factors of several chronic health conditions such as CVD, obesity, diabetes, and some cancers, and have also been linked to all-cause mortality [49–54]. However, it has been argued that their causal role in disease has been overstressed, and that they should be viewed as mediators of the adverse circumstances and psychosocial environment in which people live [55], instead of triggers of disease. Indeed, social and living conditions generating psychosocial stressors and material constraints determine whether individuals uptake harmful behaviour and whether they possess the necessary resources and motivation to care for their oral and overall health [56]. Related to this is the link between the social environment and self-perceived health and health locus of control, which in turn affects one’s ability to change harmful behaviour [56]. Therefore, while health is critically dependent on behaviour, it is necessary that behaviours are placed into context as mediators of the social, economic and psychosocial influences that are affected by stress and diminished coping abilities.

### 2.3. Immune responses to stress

With the growing realization that immune and inflammatory processes play a major role in a spectrum of chronic diseases (e.g. CVD, asthma, obesity, Alzheimer’s disease and periodontal disease) [57–61], the relationship between psychosocial stress and allostatic load, particularly in relation to immune system dysregulation, has become increasingly important. In this regard, studies have shown that stress hormones, including cortisol, epinephrine and norepinephrine, bind to specific leukocyte receptors and have deregulatory effects on their distribution and function [62]. Large population-based studies have also shown that leukocyte response to physiologic regulation by the HPA axis is altered under conditions of social isolation, eventually increasing the susceptibility to health risks. As well, chronic stress and lower SEP have been shown to suppress the phagocytic and bactericidal leukocyte functions, and to be associated with short-living leukocytes with shorter telomere lengths, a sign of cell senescence and premature ageing immunity [63, 64]. In support of this concept, functional genomic analyses have found individuals with stress-induced anxiety to have a diminished expression of gene transcripts bearing the response elements for glucocorticoids while having up-regulated gene transcripts for pro-inflammatory transcription factors [65, 66]. Genes for anti-inflammatory cytokines, such as IL-10 and IL-13 were also found to be overexpressed in response to stress [63]. These findings of both pro and anti-inflammatory cytokine expressions associated with chronic stress response have been attributed to the repeated attempts of the body to counter-balance the effects of pro-inflammation by inducing anti-inflammatory responses [63], all pertaining to the “wear and tear” concept. These cascades would obviously predispose to increased and prolonged levels of systemic and possibly local inflammation.

## Summary

Given all of the above, the relationship between social factors and immune processes in oral disease has emerged as an area of growing interest and study. Indeed, while the social patterning in oral disease has been attributed to differences in behavioural, social, economic and environmental factors, much remains underexplored in terms of how social exposures translate into biological processes. Despite the consensus that these factors are key determinants of oral disease, the psychosocial and biological pathways through which social adversity undermines oral health remains unclear and is worth investigating. As a result, we undertook a systematic review, which is presented next, to examine the plausible relationships and interactions between the social and the biological in oral disease, and to define knowledge gaps and future research opportunities in this field.

## Methods

### 4.1. Search strategy

We conducted a literature search to investigate the mechanisms linking social and psychosocial exposures to biological markers in oral disease. Published articles were obtained by searching databases, including Ovid MEDLINE, Embase, Web of Science and PsycINFO. We used the past two decades as a timeframe for our literature search (1994–2015). This timeframe was guided by the five eras of health inequalities research proposed by Adler and Stewart, where research into the underlying biological mechanisms of health inequalities started in the early 2000s [30]. The search used appropriate keywords and MESH terms (exploded) of the exposure and outcome of interest (Table 1). The search was complemented by screening the references of selected articles for studies that did not appear in the database search.

**Table 1:** Keywords, MESH terms, inclusion and exclusion criteria.

Exposure	Social, socioeconomic, psychosocial. Keywords: stress, psychosocial, psychosocial stress, psychological stress, socioeconomic status, income, education
Outcome	Oral disease, periodontitis, dental caries, changes in biological markers. Keywords: oral health, oral disease, periodont*, dental caries, tooth loss, immun*, biological markers, oral health inequalities, oral health disparities
Study Design	No restrictions on study-designs were applied
Inclusion criteria	Empirical studies, English language, human studies Stressor had to be chronic, psychosocial in nature (i.e. not physical stress) Including at least one social factors and one biological marker (e.g. immune marker)
Exclusion criteria	Non-empirical studies Studies assessing acute oral conditions, dental trauma, oral cancer

### 4.2. Study selection

To be included in this review, a study had to assess two aspects in relation to oral health outcomes: a psychosocial stressor and at least one biological marker. Studies were excluded if assessing acute, time-limited stressors (e.g. students taking examinations), physical stressors (e.g. cold, physical restraint) and if the oral health condition studied was acute, transient or self-limiting (e.g. dental trauma, recurrent herpes simplex, minor aphthous ulcers). Only articles published in the English language were considered, and no restrictions were made on the age group studied. Although a chronic oral condition, oral cancer was not the focus of this study due to the number of factors on the biological front related to its pathogenesis, which may be different than those of other oral disease generally. Finally, titles and abstracts from the initial search were reviewed to select potentially relevant articles for full review.

### 4.3. Data extraction and quality assessment

Quality assessment of the studies was carried out using the National Institute of Health (NIH) quality assessment tool for observational studies as applicable. Due to the heterogeneity of the methodologies and study designs, and as the purpose of this work was to identify potential mechanisms of social-biological interactions rather than evaluating the strength of the evidence, quantitative evaluation of the studies was not possible. Therefore, the results are presented in the narrative form.

## Results

### 5.1. Study characteristics

A total of ten studies were eligible for inclusion in this review, summarized in Table 2. The studies had different designs: four cross-sectional, four case-control and two longitudinal studies with 6-months and 5-years of follow-up, respectively. There was considerable variation amongst the studies as per population characteristics, psychosocial conditions of interest, methods of stress assessment and the covariates taken into account. Most studies investigated the relationship between psychosocial stress and periodontal disease and included a broad range of age groups (18–85 years). Dental caries in relation to psychosocial exposures was examined in two studies [67, 68]. Data from epidemiological surveys were used in four studies [68–72], whereas others recruited participants from university periodontology clinics [73], private practices [74], or through files of employees on sick leave [75]. The quality of the studies is described in Table 3.

**Table 2:** Summary of key studies exploring the relationship between structural/social, psychosocial and biological factors in oral disease.

Authors	Study design	Oral health outcome	Population	Structural/social factors	Psychosocial factors	Behavioural factors	Biological marker(s)	Conclusions
Moss et al. [71]	Case-control	Periodontitis (plaque, CAL, BOP, PD)	Participants from Erie County Risk Factor Study (71 cases, 77 controls), U.S.A	−	Daily Strains (job stain, financial strain, spouse strain, strain related to parenting children); psychological distress and coping style. Strain Scale; Brief Symptom Inventory; COPE inventory	Smoking	Antibody titres for periodontal pathogens	Depression as marker for social isolation was associated with elevated levels of antibody titres for periodontal disease at baseline and after 1-year follow-up
Giannopoulou et al. [76]	Case-control	Gingivitis, AP, EOP (PI, BOP, SUP)	Participants were selected from a private practice limited to periodontics in Athens, Greece (80)	−	Perceived stress, Modified and Perceived Stress Scale (MAPS)	Smoking	IL-1b, IL-4, IL-6 and IL-8 in the GCF	IL-4, IL-6 and IL-8 were significantly correlated with to smoking while stress was associated with IL-1b, IL-6 and IL-8 levels
Mengel et al. [73]	Case-control	AGP, ALP, CGP (GI, PI, CAL)	Patients from periodontology department, Philips-University, Marburg, Germany (40 cases; 40 controls)	−	Job-related stress, family-related stress, attitude to life. Questionnaire (non-validated)	Smoking	IL-1β, IL-6, cortisol in serum	No correlation between immunological markers, cortisol and the registered stress values. Patients with untreated AGP showed a pessimistic attitude to life and elevated serum IL-6. Sample size was too small for generalizable conclusions. Method of stress assessment was unvalidated and unstandardized
Johannsen et al.[78]	Case-control	Periodontitis (plaque, GI, CAL, PD); Number of teeth	Women on long-term sick leave for depression (20 cases, 29 controls), Stockholm, Sweden	−	Job-stress related depression (DSM-IV)	Smoking	IL-1β, IL-6, MMP-8, MMP-9	Women on long-term sick leave for depression had more plaque accumulation and higher concentrations of GCF IL-6 than controls, suggesting relationships between depressive symptoms and immune changes
Sabbah et al.[69]	Cross-sectional	Periodontitis (CAL, GB)	NHANES III (1988–1994), U.S.A	Socioeconomic position (poverty: income; education)	−	Smoking	Allostatic load	Allostatic load partly explains socioeconomic gradients in periodontal disease and ischaemic heart disease, suggesting a common stress pathway to both conditions.
Borrell et al. [70]	Cross-sectional	Periodontitis (CAL, PD)	NHANES (1999–2004), United States	Socioeconomic position (annual family income, PIR)	−	Smoking	Allostatic load	Allostatic load increases the probability of periodontitis. This association is explained by race/ethnicity.
Boyce et al. [67]	Cross-sectional	Dental Caries (dmfs, DMFS)	Kindergarten children from the Peers and Wellness Study (n = 94), East San Francisco Bay Area, California, U.S.A	Socioeconomic status (parent-reported highest household education level)	Family financial stressors (FSS)	−	Basal salivary cortisol, salivary cortisol reactivity, oral cariogenic bacteria	Low SES, higher basal salivary cortisol and larger numbers of cariogenic bacteria were each significantly and independently associated with caries. Higher salivary cortisol reactivity was associated with thinner, softer enamel surfaces in exfoliated teeth. Highest rates of dental pathology were found among children with the combination of elevated salivary cortisol expression and high counts of cariogenic bacteria.
Bakri et al. [74]	Longitudinal (6 months follow-up)	Periodontitis (PD, BOP)	45 patients with periodontitis in need of NPT, Sheffield, UK	−	Perceived stress (PSS)	Smoking	Salivary cortisol, ICTP, elastase activity in GCF	Patients under psychosocial stress had increased elastase levels and poorer outcomes following NPT
Buchwald et al. [72]	Longitudinal (5-year follow-up)	Periodontitis (CAL); Number of teeth	3300 participants from Study of Health in Pomerania (SHIP), Germany	Socioeconomic status (education, occupation, household income), marital status	−	Smoking, tooth-brushing, last dental visit	BMI, CRP	Accumulation of socioeconomic and behavioural factors augmented periodontal disease progression and systemic levels of CRP
Masterson & Sabbah [68]	Cross-sectional	Caries in children	1184 mother-child pairs usingNHANES III	Socioeconomic status (PIR)	−	Maternal care-taking behaviours	Maternal allostatic load	Maternal allostatic load is associated with caries in children and is linked to health-related maternal behaviours

**Table 3:** Quality of studies included using National Institute of Health (NIH) quality assessment tool for observational and cross-sectional studies.

Criteria	Moss et al.	Giannopoulou et al.	Mengel et al.	Johannsen et al.	Sabbah et al.	Borrell et al.	Boyce et al.	Bakri et al.	Buchwald et al.	Masterson et al.
Was the research question or objective in this paper clearly stated and appropriate?	1	1	1	1	1	1	1	1	1	1
Was the study population clearly specified and defined?	1	1	1	1	1	1	1	1	1	1
Did the authors include a sample size justification?	0	0	0	0	0	0	1	1	1	0
Were controls selected or recruited from the same or similar population that gave rise to the cases (including the same timeframe)?	1	1	1	1	NA	NA	NA	1	1	NA
Were the definitions, inclusion and exclusion criteria, algorithms or processes used to identify or select cases and controls valid, reliable, and implemented consistently across all study participants?	1	1	1	1	1	1	1	1	1	1
Were the cases clearly defined and differentiated from controls?	1	1	1	1	NA	NA	NA	1	NA	NA
If less than 100 percent of eligible cases and/or controls were selected for the study, were the cases and/or controls randomly selected from those eligible?	0	0	0	0	NA	NA	NA	0	NA	NA
Was there use of concurrent controls?	1	1	1	1	NA	NA	NA	1	NA	NA
Were the investigators able to confirm that the exposure/risk occurred prior to the development of the condition or event that defined a participant as a case?	0	0	0	0	0	0	0	0	0	0
Were the measures of exposure/risk clearly defined, valid, reliable, and implemented consistently (including the same time period) across all study participants?	1	0	0	0	1	1	1	1	1	1
Were the assessors of exposure/risk blinded to the case or control status of participants?	0	0	0	0	NA	NA	NR	1	NA	NA
Were key potential confounding variables measured and adjusted statistically in the analyses? If matching was used, did the investigators account for matching during study analysis?	1	0	1	0	1	1	0	1	1	1
1 = Yes; 0 = No; NA = not applicable, NR = not reported										

### 5.2. Oral health outcomes

Parameters used to assess periodontal disease varied between studies. In most studies, periodontal disease was assessed by measuring periodontal health parameters, including probing depth, clinical attachment loss, bleeding on probing and plaque and gingival indices [67, 69–74, 76, 77]. Dental caries was measured through decayed, missing and filled teeth/surfaces in primary and permanent teeth [68].

### 5.3. Mechanisms of social-biological interactions in oral disease

#### 5.3.1. SEP, allostatic load, and susceptibility to inflammation

Using American NHANES data, two epidemiological studies by Sabbah et al. and Borrell et al. examined the relationship between SEP (poverty: income), allostatic load and periodontal disease [69, 70]. Allostatic load was summed using measures of central obesity, blood pressure, hypertriglyceridemia, low HDL, plasma glucose, C-reactive protein (CRP) (a marker of systemic and local inflammation) and fibrinogen. Sabbah et al. found allostatic load to explain the social gradient in both periodontal disease and ischaemic heart disease. Meanwhile, Borrell et al. found the relationship between allostatic load and periodontitis to vary by race/ethnicity and attributed that to the stronger effect allostatic load may play in racial minorities who may lack coping strategies. The significance of allostatic load as outlined earlier in this review, lies in the cumulative effects of psychosocial stress that can be detrimental to different body systems, acting as a common pathway to several health conditions, including oral disease. Moreover, these effects seem to extend beyond influencing only one’s oral health. For example, in a recent study by Masterson and Sabbah, maternal allostatic load–as a measure of exposure to chronic stress–was linked to adverse care-taking behaviours, such as breast-feeding, taking a child to a dentist, and giving them breakfast daily, and was correlated to child caries experience [68]. These associations were attenuated after adjusting for SEP. While these behaviours have not been conclusively related to oral disease in children, the study highlights how the cumulative effect of chronic stress, as a result of poor living conditions, can extend to affect maternal behaviours and ultimately their children’s health.

In a longitudinal epidemiological study, Buchwald et al. found the combination of SEP (income and education) and behavioural factors (smoking) to augment the progression of periodontal disease and to be associated with higher levels of obesity and CRP. They suggested that low SEP is associated with an increased susceptibility to inflammation, and that inflammatory challenges (high levels of CRP, obesity) are most adverse in individuals with low SEP [72]. An important highlight of this study was the gender difference in disease and inflammation. Women had better periodontal health than men, in accordance with previous studies showing that women take better care of their health and smoke less than men [79]. However, while women had higher grades of education, they earned less than their male counterparts and also had higher levels of CRP, indicating an increased susceptibility to inflammation [72]. Although this study did not include psychosocial assessments, it could be hypothesized that gender inequalities may be a potential psychosocial stressor that predisposes subjects to an increased risk of inflammation.

#### 5.3.2. SEP, cortisol and pathogenic bacterial load

Boyce et al. studied the role of social disadvantage in childhood caries by assessing SEP, family financial stressors, salivary cortisol levels and the number of cariogenic bacteria in relation to dental carious lesions [67]. The study showed that low SEP, basal activation of children’s HPA axis, and higher counts of cariogenic bacteria were each significantly and independently associated with higher risk of dental caries. In fact, the highest rates of disease were exhibited amongst children with a combination of both elevated salivary cortisol levels and cariogenic bacteria. This can be explained through studies showing the ability of stress to impair sIgA production (an important player in the regulation of oral microflora) and to suppress immunity and inhibit immune cell aggregation through the release of endogenous glucocorticoids [80]. Thus, the role of stress and cortisol in contributing to an increased cariogenic bacterial load and hence the susceptibility to dental caries becomes plausible. In fact, this mechanism also applies to periodontal disease and is in accordance with previous observations by Moss et al., in which they reported increased levels of antibody titres to periodontal pathogens (*Bacteroides forsythus*) in periodontitis patients who had symptoms of depression and who experienced social isolation. Furthermore, Boyce et al. also found higher salivary cortisol reactivity to be associated with thinner, softer enamel surfaces in exfoliated teeth in children in relation to their race/ ethnicity. Previous studies have shown that glucocorticoids can induce enamel hypoplasia in children [81], affect new dentin formation and the size of the pulp chamber in animal models [82], ultimately contributing to an increased susceptibility to dental caries.

Importantly, the above observations give insight into understanding oral disease over the life-course by showing that social disadvantage can interact with biological processes to determine biological inequalities that can have long-term effects, such as the physical properties of teeth and the cariogenic bacterial load. These are eventually carried into adulthood, resulting in a long-lasting, chronic disease burden and a further contribution to inequalities in oral health.

#### 5.3.3. Stress, depression and inflammatory biomarkers

Giannopoulou et al. reported on the relationship between gingival crevicular fluid (GCF) interleukins with stress in periodontitis, where perceived stress was associated with increased levels of IL-1β, IL-6 and IL-8 [76]. It has been suggested that the high levels of cytokines in response to stress may be explained by the alteration in Th1/Th2 balance due to cortisol release, which results in a shift towards a pattern of humoral response and cytokine production by Th2 cells [83]. In a longitudinal study, Bakri et al. investigated the effects of perceived stress on the outcome of non-surgical periodontal treatment, and on markers of periodontal inflammation and bone destruction, including GCF neutrophil elastase and C-terminal telopeptide of type I collagen [74]. Their results showed that stressed patients had significantly higher elastase levels and poorer treatment outcomes following non-surgical periodontal therapy compared to the non-stressed.

An important aspect of the relationship between perceived stress and inflammatory biomarkers is understanding that stress is not the same experience for everyone and depends on a number of psychosocial factors such as coping abilities, social support and the quality of social networks [10]. In addition, one’s self-perception of stress may also relate to factors such health behaviours, and the belief of internal versus external locus of control [10]. Thus, investigating how these factors may interact and contribute to the inflammatory process is important.

Johannsen et al. investigated periodontal status in relation to interleukins and cortisol in GCF and saliva of women on long-term sick leave for depression [78]. The study found depressed women to have significantly higher levels of periodontal disease and GCF IL-6 after adjusting for age and smoking. These observations are supported by previous work that shows an association between depression and social isolation and levels of *Tannerella forsythia* in the plaque of periodontal disease patients. Meanwhile, individuals suffering depression have also been shown to be more prone to refractory periodontitis, a condition of continued disease progression and clinical attachment loss that does not correlate with plaque levels, microbiological assessments or treatment compliance, and in which hyperactive neutrophils (cells of the innate immune system) have been shown to play an important role [84, 85]. With recent studies suggesting depression to be an inflammatory condition characterized by activated cell-mediated immunity and immune cell glucocorticoid resistance [86, 87], again, a possible common stress pathway can potentially be at play in the pathogenesis of both conditions.

#### 5.3.4. Health behaviours

A number of studies included in this review have shown that the relationship between social and psychosocial factors, inflammatory biomarkers and periodontal disease can be explained by smoking, indicating that behaviour is a link through which social and psychosocial conditions can lead to oral disease. This corresponds to studies that have shown behaviours to partly explain the social gradient in oral health [88], and goes with the notion that behaviours are psychosocial in nature in the sense that they are responses to psychosocial stressors and adverse circumstances, and should therefore be considered as mediators of oral disease.

Yet a few studies have found health behaviours (e.g. smoking) to not explain the relationship between SEP and inflammatory biomarkers [72] or depression and inflammation [78]. This suggests that, behaviours, although considered as mediators of stress, are not the only pathway through which stress translates into disease, further suggesting a direct pathway through which social factors can affect biological processes, independent of health behaviours.

### 5.4. Conceptualizing social-biological interactions in oral disease

**Fig 1:**
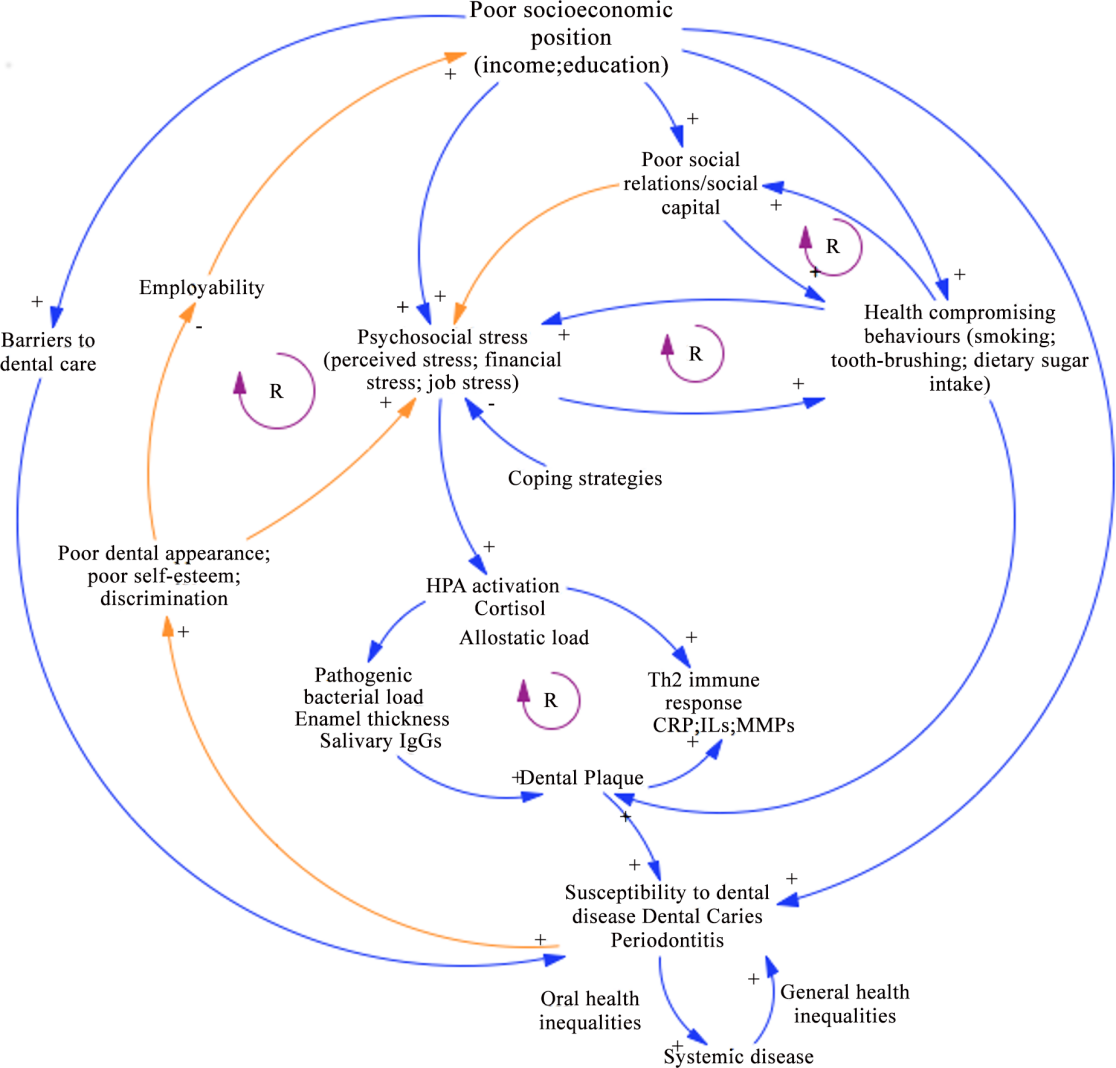
Dynamic conceptual model of social-biological interactions. The model demonstrates the interdependent relationships between different variables involved in oral disease. Blue lines are relationships derived from studies in Table 2. Orange lines represent hypothetical relationships. (R): reinforcing loops; signs (+/-) on arrowheads: polarity of the relationship between variables.

Despite the plethora of evidence on the social and psychosocial influences on oral health outcomes, only a few empirical studies were found that have considered the social causes of stress and their potential effects on the pathophysiology of oral disease. Some difficulty was encountered in arriving at the specific mechanism(s) of social-biological interactions due to a number of factors. First, is the complex nature of the topic and the multiple variates and co-variates that may be at play in the development and progression of oral disease. To be sure, the populations examined in the studies varied from groups in population surveys to individuals recruited at university clinics. Different types of social stressors where studied varying between job-stress, family stress and financial stress. Also, the method of stress assessment varied between psychometric and biochemical methods. This diversity made it difficult to compare results between studies. However, some putative mechanisms of social-biological interactions can be abstracted from the summative results of this review, which we have plotted as a dynamic conceptual model of the plausible social-biological interactions (Fig 1). In addition, some links in the model are derived from the literature on the social determinants of oral and general health, and have not been included in this review.

Conceptualizing the social causes of oral disease has been robustly discussed in the literature with several theoretical explanations, varying in their emphasis and standpoint, ranging from materialist, behavioural, psychosocial and life-course perspectives [2, 89–91]. Several mega-models have depicted the relationships between social factors and health, and have provided the theoretical basis for much of the work on the social determinants of oral and general health [56, 92–95]. These models initiated a paradigm of integration between the social and biological determinants of health, elaborating on how biological pathways exist in a social and psychosocial context. Indeed, the models not only provide an understanding of how potential causal factors may interrelate, but also serve as guides to points of intervention. However, many of these models depict the social factors as distal antecedents to the more proximal biological causes of disease and do not elaborate on the complexity of the dynamic and reciprocal relationships that potentially exist between social and biological factors. In spite of their contribution to policy-making, models that conceptualize social factors as antecedents to biological factors without considering the multiple interactions that may exist between them may have created and/or promoted a false dichotomy between the social and the biological, and may have indirectly given biomedical researchers and clinicians the notion that it may be possible to ignore the social factors and intervene only at the level of the proximal causes of disease. Thus, despite repeated calls to incorporate social causes into health research, social antecedents models might not change how biomedical research is conducted and how biological questions are framed and answered [96].

Therefore, understanding social-biological interactions in oral disease requires a view of the social as intertwined with the biological. Here, we propose a conceptual model that illustrates the dynamicity and reciprocity between the different social and biological factors in oral disease, and that shows disease as the result of complex interactions between molecular, biological and social systems with positive and negative feedback loops [97].

Dynamic models depicting such complex relationships have been previously applied to epidemiology to understand the mechanisms of disease including work on diabetes and CVD, and have been employed in understanding cellular and molecular pathways of disease [98–100]. Our model demonstrates the potential putative interdependencies between the different factors involved in oral disease, illustrating that psychosocial stress results from and affects socioeconomic conditions and the social and work environment, eventually leading to behavioural and biological changes that compromise the body’s protective mechanisms against oral disease. Under this model of social-biological interactions, social and psychosocial experiences have the capacity to directly alter both the structure and function of biological systems. Thus, these factors are not viewed as distinct and susceptible to separation but rather as integral parts of a complex system that ranges from epigenetic and cellular reactions to structural factors, where the social environment is a key piece in understanding the function of the system [96]. The dynamic loops in the model facilitate deriving empirically testable relationships. It is important to note, however, that the purpose of the model is not to include all geopolitical and structural determinants of oral health, but to provide an understanding of the possible interactive relationships between social adversity and pathobiological processes in oral disease. Therefore, more variables, relationships and dynamic loops can be added onto the model as interdisciplinary research in this area advances.

## Policy Implications and Research Opportunities

People live in political, social and economic systems that shape their access to resources, behaviour and biology. Understanding the pathways through which these factors interplay to shape oral health inequalities can help inform public health policies about the causal pathways to oral disease and can therefore lead to a much needed paradigm shift towards public health interventions at the level of the fundamental conditions that put people at *“risk of risks”* − as described by Link and Phelan [101]. However, unraveling these pathways requires refuting the existing false dichotomies between the social and the biological, and developing research approaches that give equal weight to *both* the social on the one hand, and the biological on the other.

In a critical review of theories in social epidemiology, Krieger suggests that theories and research approaches are needed that recast differences in biological outcomes as embodied expressions of modifiable social exposures [102]. In that regard, research on the social-biological interactions in oral disease represents a fertile field not only to identify social and biological risk factors, but also to recognize the interplay between social exposure, susceptibility and resistance. Ultimately, this can help identify those at higher risk for developing oral disease due to adverse social circumstances, and prevent and/or control such disease experience.

While addressing the social and living conditions in which we live has arguably been hindered by weak policies, bias to downstream approaches and a lack of political will, understanding the ‘biology of social adversity’ and identifying how social and material factors become incorporated within biology is integral to developing evidence-informed arguments on the social origin of disease. This is particularly important if we are to devise new and novel interventions that address the social determinants of health and tackle oral health inequalities.
